# Expanded memory CD4+ T Cells in the fetal and the infant Gut; a mucosal route for mother-to-child transmission of HIV-1

**DOI:** 10.1186/1742-4690-9-S2-O21

**Published:** 2012-09-13

**Authors:** MJ Bunders, C van der Loos, PL Klarenbeek, J van Hamme, J Wilde, N de Vries, RA van Lier, N Kootstra, ST Pals, TW Kuijpers

**Affiliations:** 1 Academic Medical Centre, Amsterdam, the Netherlands

## Background

Cord blood-derived CD4+ T cells have a naïve phenotype and do not express CCR5, the mandatory co-receptor for transmitted HIV-1 R5 strains in infants. This leaves the question unanswered: what are the target cells for MTCT of HIV-1 and where do they reside? We hypothesized that in infant mucosal tissues, CD4+CCR5+ T cells may be present to facilitate mucosal transmission of HIV-1.

## Methods

Using multicolor immuno-histochemistry, flowcytometry and next-generation sequencing of the T cell receptor, we analyzed various human fetal and infant tissues to identify memory CD4+ T cells as targets for HIV-1.

## Results

Here, we demonstrate the previously unrecognized abundance of memory CD4+CCR5+ T cells in the human fetal and infant gut mucosa. CD4+ T cells from mesenteric lymph node were mostly naïve, similar to blood. T helper differentiation profiles as determined by transcription factors differed by tissue, with T-bet and RORγt predominantly expressed by memory T cells in the gut mucosa. Next-generation sequencing for high-resolution screening of the T-cell receptor β-chain repertoire of clonal T cells as a hallmark of memory cells, identified expanded T cell clones in the gut mucosa (30%) and not in lymph node or cord blood. The gut mucosal fetal and infant CD4+ T cells were extremely susceptible to HIV-1 without any prestimulation; pol proviral DNA levels were similar to infected PHA stimulated adult PBMCs.

## Conclusion

In conclusion, we show that extensive adaptive immunity, with a tissue-depended distribution is present before birth, resulting in the gut mucosa as the preferential site for memory CD4+ T cells. These memory CD4+CCR5+T cells provide a large pool of susceptible cells for ingested HIV-1 at birth and during breastfeeding, indicating a mucosal route of MTCT of HIV-1, which can be targeted in future prevention strategies.

